# Genomic Comparison of Conjugative Plasmids from *Salmonella enterica* and *Escherichia coli* Encoding Beta-Lactamases and Capable of Mobilizing Kanamycin Resistance Col-like Plasmids

**DOI:** 10.3390/microorganisms9112205

**Published:** 2021-10-23

**Authors:** Elizabeth A. McMillan, Ly-Huong T. Nguyen, Lari M. Hiott, Poonam Sharma, Charlene R. Jackson, Jonathan G. Frye, Chin-Yi Chen

**Affiliations:** 1 U.S. Department of Agriculture, Agricultural Research Service, U.S. National Poultry Research Center, Bacterial Epidemiology and Antimicrobial Resistance Research Unit, Athens, GA 30605, USA; elizabeth.mcmillan@usda.gov (E.A.M.); lari.hiott@usda.gov (L.M.H.); charlene.jackson@usda.gov (C.R.J.); 2 U.S. Department of Agriculture, Agricultural Research Service, Eastern Regional Research Center, Molecular Characterization of Foodborne Pathogens Research Unit, Wyndmoor, PA 19038, USA; ly.nguyen@usda.gov (L.-H.T.N.); chin-yi.chen@usda.gov (C.-Y.C.); 3 Institute of Biosecurity and Microbial Forensics, Department of Biochemistry and Molecular Biology, Oklahoma State University, Stillwater, OK 74078, USA; poonam.sharma@okstate.edu

**Keywords:** plasmids, conjugation, *Salmonella*, *E. coli*

## Abstract

*Salmonella enterica* and *Escherichia coli* are important human pathogens that frequently contain plasmids, both large and small, carrying antibiotic resistance genes. Large conjugative plasmids are known to mobilize small Col plasmids, but less is known about the specificity of mobilization. In the current study, six *S. enterica* and four *E. coli* strains containing large plasmids were tested for their ability to mobilize three different kanamycin resistance Col plasmids (KanR plasmids). Large conjugative plasmids from five isolates, four *S. enterica* and one *E. coli*, were able to mobilize KanR plasmids of various types. Plasmids capable of mobilizing the KanR plasmids were either IncI1 or IncX, while IncI1 and IncX plasmids with no evidence of conjugation had disrupted transfer regions. Conjugative plasmids of similar types mobilized similar KanR plasmids, but not all conjugative plasmid types were capable of mobilizing all of the KanR plasmids. These data describe some of the complexities and specificities of individual small plasmid mobilization.

## 1. Introduction

Both *Salmonella enterica* and *Escherichia coli* are common causes of foodborne illness in the United States and globally [1]. Combined, *Salmonella* and Shiga-toxin producing *E. coli* were responsible for 23.4% of reported foodborne illnesses in 2019 at sites participating in FoodNet in the U.S. [2]. Unfortunately, an increasing number of these infections display resistance to antibiotics [3]. Many of the genes conferring these resistances are carried by plasmids, both large and small, which could increase the mobility of these genes and by extension increase the spread of antibiotic resistance [4]. For example, β-lactamase genes, *bla*_CMY-2_ in particular, have been shown to frequently be carried by large plasmids especially of types IncA/C and IncI1 in *Salmonella* [5].

While many large plasmids are able to transfer to new cells independently via conjugation, not all plasmids are self-transmissible. Mobilizable plasmids are those that cannot transfer via conjugation on their own, but require a “helper plasmid” possessing the necessary genes to encode the mate-pair structures which mobilizable plasmids can utilize to transfer to a new cell [6]. Mobilizable plasmids may utilize their own relaxase/nikase or those present on the conjugative helper plasmid [7,8]. Many large plasmids, such as types IncF, IncP, and IncI1, have proven capable of mobilizing small plasmids [9]. However, less attention has been given to the differences in mobilization capabilities of these large conjugative plasmids.

Previously, small ColE-like plasmids carrying kanamycin resistance *aph(3′)*-I genes were categorized based on their plasmid replication/maintenance region and mobilization gene(s) [10,11,12,13,14]. In order to investigate the specifics of mobilization of these KanR plasmids, we screened a collection of β-lactam resistant *Salmonella* and *E. coli* strains for large plasmids and the ability of those large plasmids to mobilize varying KanR plasmids in tri-parental mating experiments. For isolates producing transconjugants, whole genome sequencing and further comparison of the plasmid sequences were completed.

## 2. Materials and Methods

### 2.1. Strains

To maximize potential for presence of large plasmids, β-lactam-resistant, KAN STR-susceptible *Salmonella enterica* (*n* = 6) and *Escherichia coli* (*n* = 4) isolates were selected from a collection of multidrug resistant isolates of animal origin (Table 1). Isolates were tested for their ability to convert *E. coli* NEB10β to AMP-resistant via conjugation overnight. The plasmid replicon types and the β-lactamases present in the original isolates and their transconjugants were characterized using a PCR-based plasmid replicon typing kit (PBRT; Diatheva, Cartoceto, Italy) and the ARM-D β-lactamases ID kit (Streck, La Vista, NE, USA), respectively. The transconjugants were screened for the absence of small plasmids by extracting DNA using QIAprep Spin Miniprep kit (Qiagen, Germantown, MD, USA), and clones without visible small plasmids were chosen for further testing (described below) for their ability to mobilize different ColE-like kanamycin resistance plasmids (KanR plasmids) [10,12,13]. Of those, three isolates were selected for sequencing (*n* = 2 *S. enterica*, *n* = 1 *E. coli*) and one isolate, strain five (CRJJGF_00030), was sequenced and characterized as a part of a previous study [15]. A transformant containing the I1 plasmid of isolate #2 and the transconjugants of isolates #3, #5, and #12 were also sequenced.

### 2.2. KanR Plasmid Mobilization Tests on Agar

The ability of the conjugative plasmids to mobilize different KanR plasmids (pU302S [10], pSN11/00Kan [12], and pSe-Kan [13]) was tested using tri-parental mating on agar for qualitative assessment. Cultures of donor (NEB5α carrying KanR plasmid; KAN^R^), recipient CAG18483 (TET^R^; F-, *fad*L771:Tn10, obtained from Coli Genetic Stock Center, #7407), and helper (NEB10β carrying conjugative plasmid; AMP^R^) were adjusted to ~10^8^ CFU/mL and mixed at the ratio of 1:5:1 (10 μL:50 μL:10 μL) on a LB agar plate, and incubated at 37 °C for six hours. Mating mixtures were then resuspended into LB broth and plated on LB agar supplemented with kanamycin A (50 mg/L) and tetracycline-HCl (2 mg/L) for selection. Plates were incubated overnight at 37 °C and the KanR plasmid mobilization was scored as positive or negative.

### 2.3. Sequencing

Strains were sequenced using Nextera XT library kits on an Illumina MiSeq. Raw reads were assembled with A5 [16]. Plasmid replicons were identified using PlasmidFinder (https://cge.cbs.dtu.dk/services/PlasmidFinder/; accessed on 31 August 2020) and plasmid contigs were identified using BLAST in comparison to known plasmid sequences in the non-redundant database [17]. Plasmid sequences were annotated using RAST and gene assignments were confirmed using BLAST in comparison to the non-redundant protein database [18]. Antibiotic resistance genes were identified using ResFinder (https://cge.cbs.dtu.dk/services/ResFinder/; accessed on 31 August 2020) [19]. The *ori*T sequences were identified with oriTFinder [20]. For plasmids where oriTFinder could not identify an *ori*T sequence, the *ori*T was manually annotated based on the homology with the *ori*T of IncX1 plasmid pOLA52 (NC_010378.1) [21]. Plasmid maps were generated using SnapGene (https://www.snapgene.com/; version 4.0.8). The transfer regions of each plasmid were extracted and aligned with the transfer regions of plasmids of similar type within this study as well as the prototypical plasmid of their incompatibility group using Mauve [22]. Prototypical plasmids used were IncX2 plasmid R6K (http://www.sanger.ac.uk/Projects/Plasmids/; accessed on 31 September 2020) and IncI1 plasmid R64 (AP005147.1) [23]. The transfer region for p3I1 could not be aligned because the sequence was discontinuous. Plasmids were compared with the matching transconjugant plasmid using BLAST. The Blast Ring Image Generator (BRIG) was used to generate ring comparison figures [24].

## 3. Results

A total of 10 isolates were investigated, each of which contained at least one large plasmid. All 10 strains also contained a detectable β-lactamase gene, *bla*_CMY-2_. Seven of these strains yielded transconjugants in the bi-parental mating: all *S. enterica* strains investigated, but only one *E. coli* strain. Only plasmids of replicon types I1, X1, and X4 showed evidence of conjugation. However, not all plasmids of these types yielded transconjugants. With the exception of one isolate (strain #3), *bla*_CMY-2_ was the β-lactamase gene transferred to the recipient cell (Table 1).

Seven strains were subjected to whole genome sequencing with an average of 252 contigs per sequence. Plasmid replicon genes and antibiotic resistance genes were identified in every sequence (Table 1). Plasmids identified in original strains were named for the isolate number and replicon type. Plasmids in transconjugants were given the same name with a TC designation (Table 2). In each isolate/transconjugant pair, only one large plasmid containing a β-lactamase gene transferred. However, three of four isolates contained an additional large plasmid that did not transfer (Table 1). Plasmids of IncI1 or IncX type were assembled regardless of whether they transferred or not (Table 2, Supplementary Figures S1 and S2). The transfer regions of both IncI1 plasmids that did not transfer were disrupted by transposons or other sequences (Supplementary Figures S1 and S2). Isolates #2, #3, and LCp2 contained a ColVC plasmid approximately 2 kb in size; the ColVC is partial (~one kb) in TC5f (Table 1).

Plasmids from original isolates were compared to plasmids in transconjugant clones. Plasmids p3X1 and p3X1-TC contained identical sequence with no SNP differences. A 163 bp insertion in a non-coding region of p3X1-TC was determined to be an assembly artifact rather than sequence change; this sequence was also present in the whole genome sequence of the original isolate, but on a small contig that could not be joined to the plasmid. p5I1 and p5I1-TC were identical. p12X4-TC contained all the sequence within p12X4 but had two insertions not contained within the genomic sequence of the original isolate: a 112 bp insertion in a non-coding region and a 275 bp insertion overlapping a non-coding region and transposase gene. p2I1 and the retransformed p2I1-LC contained the same sequence, except for a 258 bp deletion between two genes in the transfer region of p2I1-LC and a 289 bp deletion between two genes outside of the transfer region. However, there were also minor rearrangements related to assembly artifacts and less than 10 mismatches.

**Figure 1:**
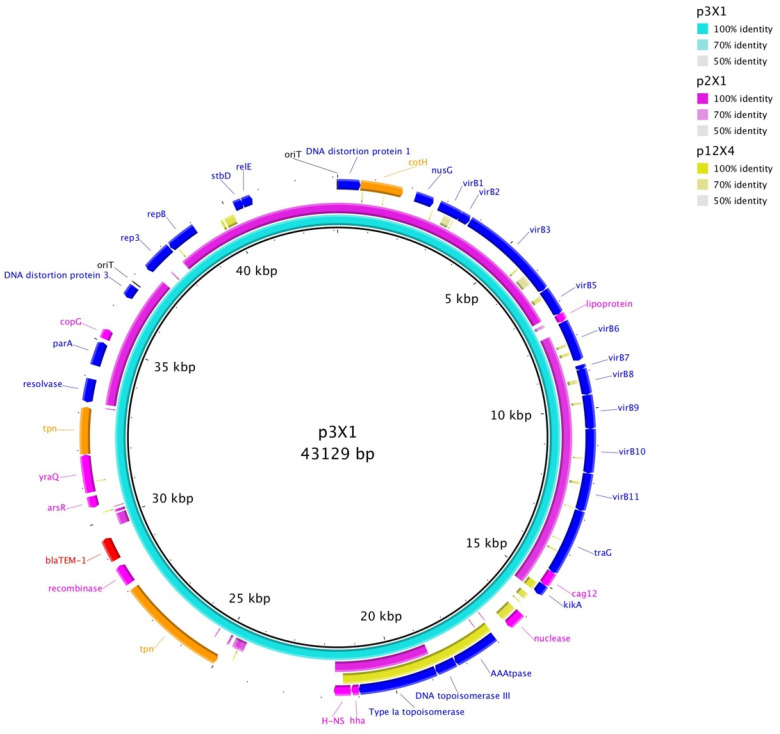
Blast comparison of the IncX plasmids.

**Figure 2:**
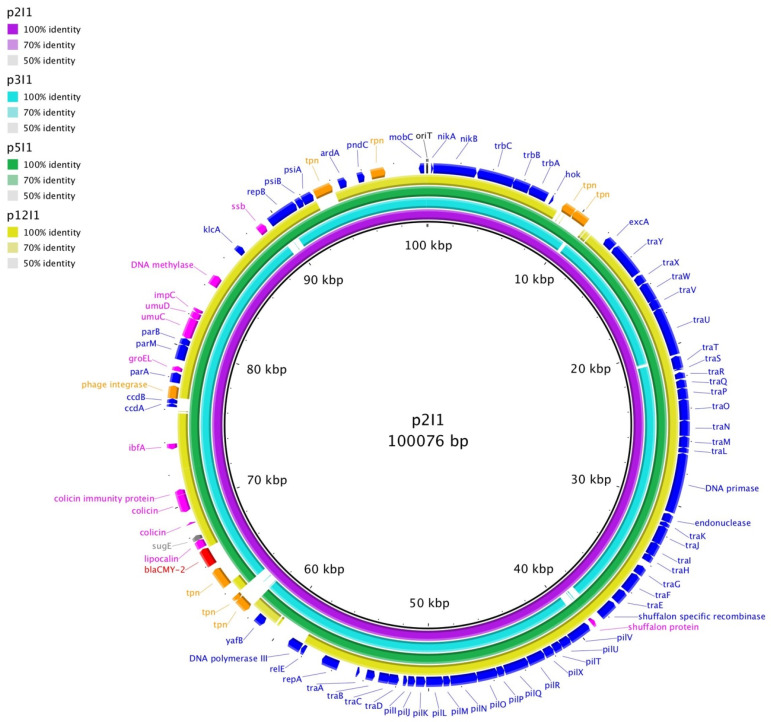
Blast comparison of IncI1 plasmids.

Alignments were generated comparing the transfer region of each plasmid of similar Inc type to each other and to a prototypical plasmid of each type. Plasmids were also compared to the plasmid of similar type that did not transfer (Figure 1 and Figure 2; Supplementary Figures S3 and S4). IncX1 plasmids showed more similarity to each other than to the IncX4 plasmid or the prototypical IncX plasmid, which is IncX2 (Figure 1; Supplementary Figure S3). IncI1 plasmids showed homology in their transfer regions, except p12I1, which contained both an insertion and deletion (Figure 2; Supplementary Figure S4).

Conjugative plasmids from at least five of the 10 original isolates were capable of mobilizing small plasmids (Table 3), four of which were sequenced: p2I1, p3X1, p5I1, and p12X4 (and those in the corresponding transconjugant/transformant p2I1-LC, p3X1-TC, p5I1-TC, and p12X4-TC). These plasmids were able to mobilize the KanR plasmids investigated with varying success. Plasmids of the same Inc type were able to mobilize the same small plasmids. pSN11/00Kan, which carries the *mob*C-*mob*ABD operon, can be mobilized by all conjugative plasmids tested (IncI1, X1, and X4). Neither IncX plasmid could mobilize plasmids pU302S (carries *nikA*) or pSe-Kan (does not carry known *mob* genes), which suggests the Col plasmid-borne *mob* genes are required for IncX assisted plasmid mobilization (Table 3).

## 4. Discussion

Plasmids and β-lactamase genes were identified in 10 isolates (*n* = 6 *S. enterica* and *n* = 4 *E. coli*). We then determined the ability of the large plasmids in each isolate to mobilize small KanR plasmids. In all but one case, only one large plasmid transferred via conjugation (selected for AMP resistance) in each isolate regardless of the number of large plasmids present in each isolate. KanR plasmids were mobilized with varying success, depending on plasmid type.

Here we showed that pU302S, which carried just *oriT* and a *nikA* accessory protein, can be mobilized by IncI1 (p2I1-LC and p5I1-TC). This provided the only other experimental evidence than that of NTP16 that this group of diverse plasmids which lacks a relaxase may be mobilized by the IncI family [25]. While mobilization of plasmids lacking a relaxase has been documented, the essential genes necessary for transfer, which are provided by a conjugative plasmid, have not been specified yet. IncI1 plasmids also mobilized pSN11/00Kan, the KanR plasmid carrying the *mobC*-*mobABD* mobilization region, as well as the pSe-Kan which possesses two different *oriT* regions but no other identifiable mobilization accessory protein.

Plasmids from original isolates were nearly identical to their transconjugants. While there were minor differences in the sequences of these plasmids, it is most likely that these differences are related to sequencing and/or assembly error, rather than genetic change. Sequencing and assembly can introduce errors into the final sequence, especially in repetitive elements [26]. Further, the insertions and deletions detected were present near the beginning and ends of contigs and less than the average length of a paired end read. Long-read sequences combined with the short reads collected here would help resolve these discrepancies.

Both IncI1 plasmids that did not transfer had disrupted transfer regions. In p12I1, the transfer region was disrupted by a partial *bla*_CMY-2_ gene, the insertion occurring where *tra*G is located in the other IncI1 plasmids investigated. In p3I1, the transfer region was disrupted in several places by transposons: inserted between the *tra*T and *tra*U genes, between *tra*M and *tra*N, and inside the DNA primase. It was not possible to close the assembly of p3I1 because there was no overlap of contig ends, possibly because of insertion of these transposons and repetitive elements, which can be difficult to sequence. In contrast, the IncX1 plasmid that did not transfer (p2X1) had complete transfer regions similar to p3X1, which was conjugative. It is possible that this plasmid is conjugative but either did not transfer, or transconjugants containing this plasmid were not selected because the plasmid did not contain a β-lactamase gene.

Isolates #2 and #3 contained a 2-kb ColVC plasmid. The ColVC plasmids in the two isolates were identical and appeared to be transferrable. All the transconjugants derived from isolate #2 carried additional small plasmid(s), as well as the sequenced transformant, LCp2. While the isolate #5 sequence did not contain a ColVC plasmid, transconjugant clone TC5f did. Isolate #5 was sequenced as part of another study using a different sequencing technology several years prior to this investigation. It is possible that the plasmid was not detected because the conjugation experiments were not conducted with the same culture (different retrieval from frozen storage) or that the sequencing technology did not detect the plasmid at the time of sequencing. Considering that only those transconjugant clones without visible small plasmid bands were selected for further investigation, it was unexpected to find that some TC clones still possessed ColVC plasmids within their whole genome sequence. Possible explanations for this discrepancy between sequencing and wet lab results could be (1) the ColVC plasmid may be present in lower copy number than other small plasmids, resulting in more varied miniprep yield between different samples that sometimes failed to be visualized on the gel; (2) uncut, supercoiled plasmids tend to run as smears, not sharp bands, on agarose gel, and do not take up DNA intercalating dyes easily, thus further reducing detection sensitivity. None of the ColVC plasmids contained any antibiotic resistance genes but their presence and the ability to be co-transferred does highlight the relevance of Col plasmids in natural environments.

In conclusion, similar types of large conjugative plasmids were able to mobilize the same (sub)group of KanR plasmids. Plasmids with disrupted transfer regions were not able to conjugate. We were able to show that the *nikA*-carrying KanR plasmid can be mobilized by IncI1 plasmids but not the IncX plasmids. Further studies with additional replicon types are needed to understand the full range of large plasmids capable of mobilizing these and other Col plasmids and will be important in understanding the transfer risk of a given antibiotic resistance gene.

## Figures and Tables

**Table 1:** Strain characterization: antibiogram, plasmid replicon typing, and β-lactamase gene.

Original Isolate							Neb10β Transconjugant			
Strain	Organism	Isolation Source	Resistance Profile ^a^	Replicon Type (PBRT) ^b^ [Sequencing]	*bla* Gene ^c^	NGS ^d^	Clone ID ^e^	Replicon Type (PBRT) [Sequencing]	*bla* Gene ^c^ [Sequencing]	KanR Plasmid Mobilization ^f^
1	*S. enterica* Infantis	Cattle	AMP AUG AXO CEP FOX TIO	I1	CMY-2		TC1d	I1	CMY-2	N.D.
2	*S. enterica* Heidelberg	Cattle	AMP AUG AXO CEP FOX TIO	I1, X1 [ColVC]	CMY-2	√	LCp2 *	I1 [ColVC]	CMY-2	All
3	*S. enterica* Typhimurium var. 5-	Cattle	AMP AUG AXO CEP FOX TIO	I1, FIB, X1 [FIC, ColVC]	CMY-2	√	TC3c	X1	Unk [TEM-1]	pSN11/00Kan
4	*S. enterica* Typhimurium	Cattle	AMP AUG AXO CEP FOX TIO	I1, FIIS	CMY-2		TC4d	I1	CMY-2	All
5	*S. enterica* Infantis	Chicken	AMP AUG AXO FOX TIO	I1	CMY-2	√	TC5f	I1 [ColVC]	CMY-2	All
6	*S. enterica* Saintpaul	Turkey	AMP AUG AXO FOX TIO	I1, X1	CMY-2		TC6d	I1, X1	CMY-2	N.D.
7	*E. coli*	Chicken	AMP AUG AXO COT FIS FOX GEN NAL TIO	I1, FIB, I2, K	CMY-2		N.A.			
8	*E. coli*	Chicken	AMP AUG AXO FOX GEN TIO	FIB, B/O, K	CMY-2		N.A.			
10	*E. coli*	Chicken	AMP AUG AXO FIS FOX GEN TIO	I1, FIB, X1, B/O, K	CMY-2		N.A.			
12	*E. coli*	Dog	AMP AUG AXO CIP FOX GEN NAL TIO	I1 [X4, FIA, FIB, FII, Col]	CMY-2	√	TC12d	Unk [X4]	CMY-2	pSN11/00Kan

^a^ Abbreviations: Ampicillin (AMP), Amoxicillin-clavulanic acid (AUG), Ceftriaxone (AXO), Cephalothin (CEP), Chloramphenicol (CHL), Ciprofloxacin (CIP), Sulfamethoxazole-trimethoprim (COT), Cefoxitin (FOX), Sulfisoxazole (FIS), Gentamicin (GEN), Nalidixic acid (NAL), Ceftiofur (TIO), Streptomycin (STR), Unknown (Unk). ^b^ Determined using PCR-Based Plasmid Replicon Typing kit (Diatheva). Unknown, not positive with any of the primers; additional replicons identified using sequence result shown between square brackets. ^c^ Identified using ARM-D β-lactamase ID kit (Streck). Unknown, not positive with any of the primers; sequence result shown between square brackets. ^d^ Strains (transconjugant clone and the corresponding original isolate) selected for sequencing are indicated by “√”. ^e^ N.A., transconjugant not available; *, all transconjugants showed the presence of 1 or 2 small plasmid(s); LCp2 was generated from transformation of the Large-construct plasmid prep (Qiagen) and screened for the lack of visible small plasmid band. ^f^ via tri-parental mating; N.D., not determined; All, pU302S, pSN11/00Kan, and pSe-Kan.

**Table 2:** Sequenced plasmids information.

Plasmid	Replicon Type	Size ^a^	AR Genes	Conjugated?
p2I1	I1	100kb	*bla*_CMY-2_	Yes
p2I1-LC ^b^	I1	100kb	*bla*_CMY-2_	Yes ^c^
p2X1	X1	38kb	N/A	No
p3X1	X1	43kb	*bla*_TEM-1_	Yes
p3X1-TC	X1	43kb	*bla*_TEM-1_	Yes (Transconjugant)
p3I1	I1	101kb	*bla*_CMY-2_	No
p5I1	I1	99kb	*bla*_CMY-2_	Yes
p5I1-TC	I1	99kb	*bla*_CMY-2_	Yes (Transconjugant)
p12X4	X4	37kb	*bla*_CMY-2_	Yes
p12X4-TC	X4	37kb	*bla*_CMY-2_	Yes (Transconjugant)
p12I1	I1	94kb	N/A	No

^a^ Size is approximate to the nearest whole number. ^b^ LC = large construct. ^c^ Transformant was used for the sequencing and characterization.

**Table 3:** Qualitative assessment of KanR plasmid mobilization using tri-parental agar mating. + indicates donor plasmid was mobilized, − indicates donor plasmid was not mobilized.

Donor Plasmid	Mobilization Genes	Helper Strain				
		LCp2	TC3c	TC4d	TC5f	TC12d
pU302S	*nikA*	+	−	+	+	−
pSN11/00Kan	*mobC-mobABD*	+	+	+	+	+
pSe-Kan	Unknown	+	−	+	+	−

## Data Availability

Sequences generated as a result of this study can be accessed via NCBI under BioProject number PRJNA756294.

## Supplementary Materials

The following are available online at https://www.mdpi.com/article/10.3390/microorganisms9112205/s1, Figure S1: IncI1 plasmids investigated, Figure S2: IncX plasmids investigated, Figure S3: progressiveMauve alignment of the transfer regions of R6K, p2X1, p3X1-TC, and p12X4-TC, Figure S4: progressiveMauve alignment of the transfer regions of R64, p2I1, p5I1-TC, and p12I1.
