# Supplementary material for: Genomic Comparison of Conjugative Plasmids from Salmonella enterica and Escherichia coli Encoding Beta-Lactamases and Capable of Mobilizing Kanamycin Resistance Col-like Plasmids
Source: Microorganisms. 2021 Oct 23;9(11):2205. doi: 10.3390/microorganisms9112205 (PMC8623487; doi:10.3390/microorganisms9112205)
Supplement: Supplementary file 1 [file microorganisms-09-02205-s001.zip › microorganisms-1422358-supplementary.pdf]

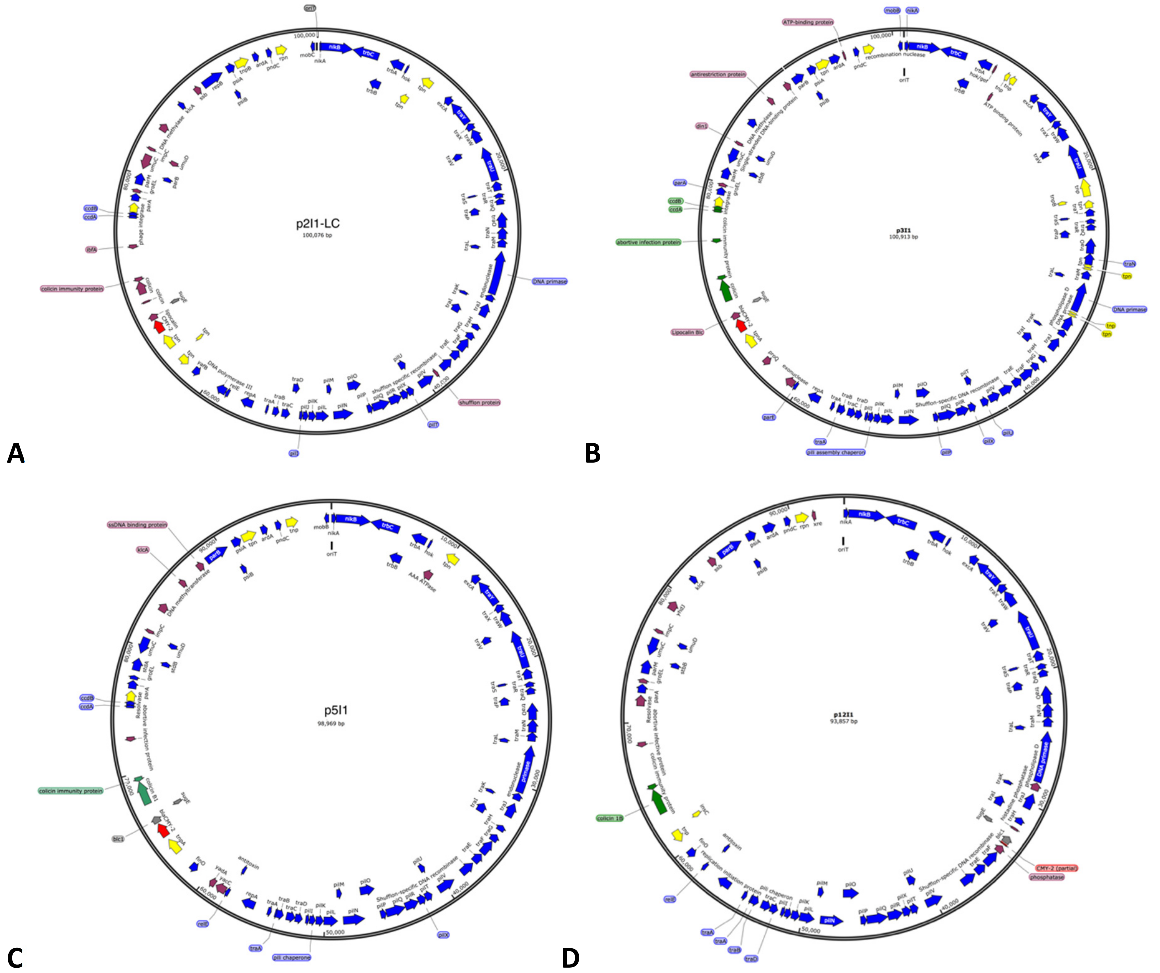

Supplementary Figure S1. IncI1 plasmids investigated. A. p2I1-LC. B. p3I1 (contigs arranged according to p5I1). C. p5I1. D. p12I1.

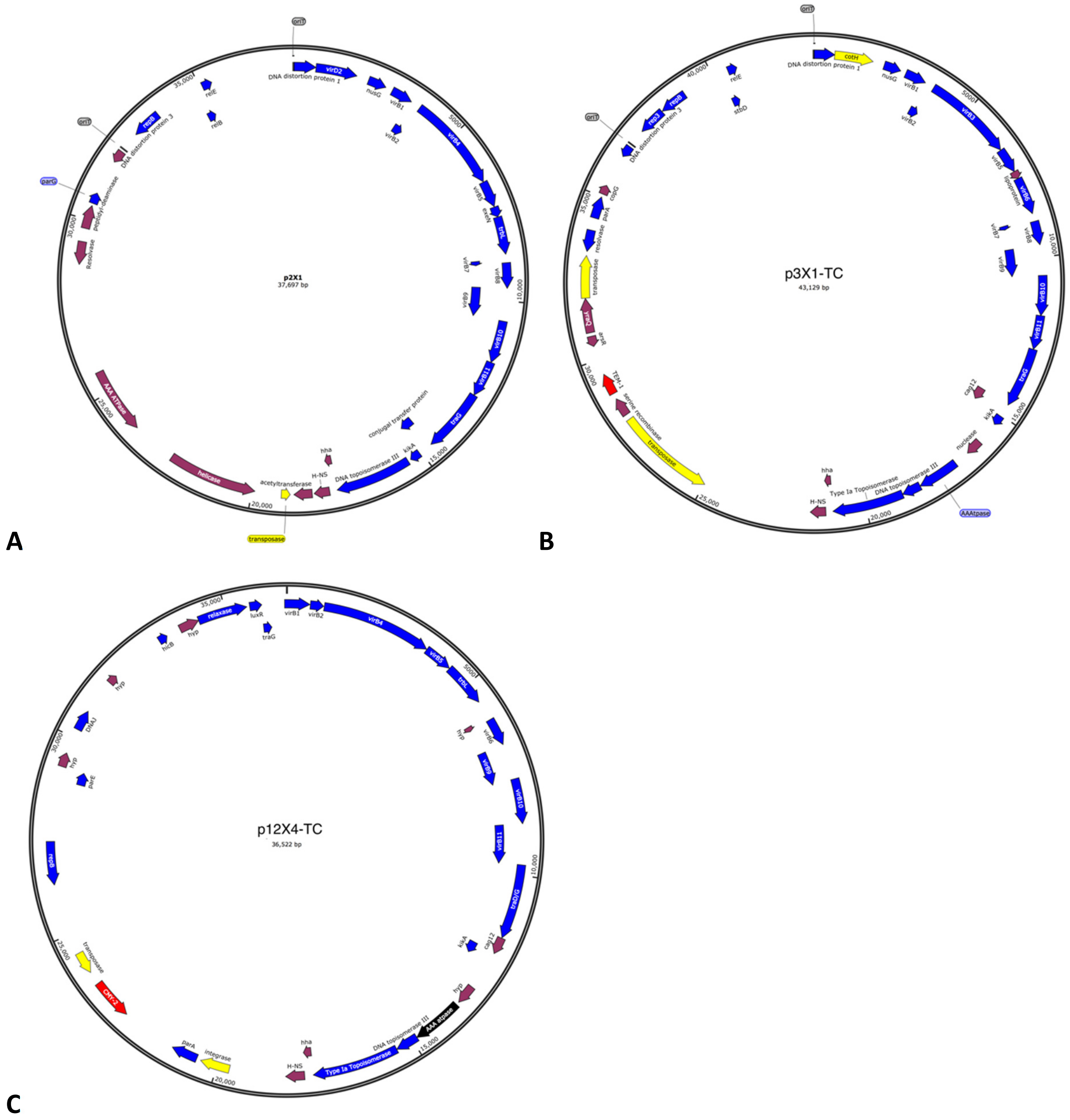

Supplementary Figure S2. IncX plasmids investigated. A. p2X1. B. p3X1-TC. C. p12X4-TC.

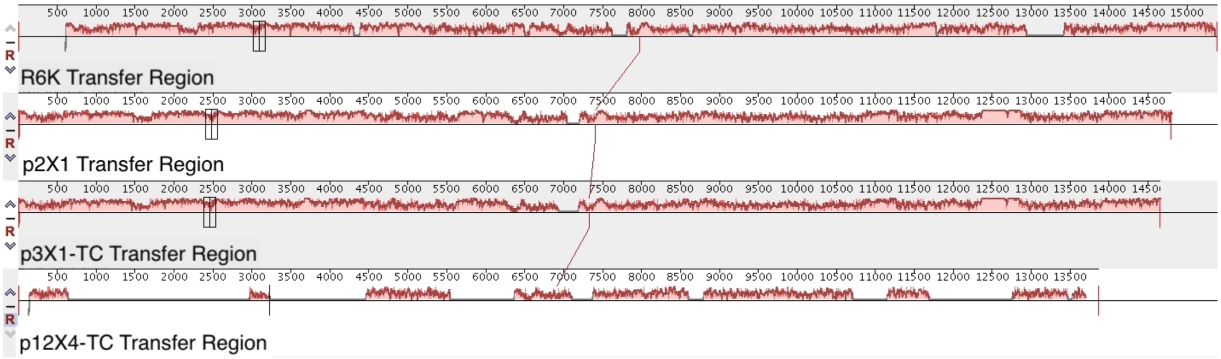

Supplementary Figure S3. progressiveMauve alignment of the transfer regions of R6K, p2X1, p3X1-TC, and p12X4-TC.

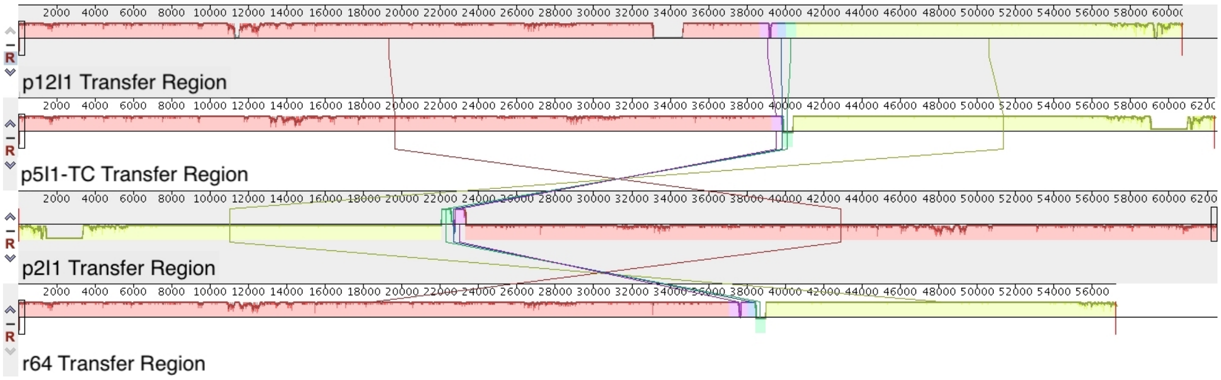

Supplementary Figure S4. progressiveMauve alignment of the transfer regions of R64, p2I1, p5I1-TC, and p12I1.
